# Integrated transcriptomic analysis identifies lactylation-linked gemcitabine resistance and therapeutic targets in intrahepatic cholangiocarcinoma

**DOI:** 10.3389/fcell.2025.1611434

**Published:** 2025-09-01

**Authors:** Wenwei Xie, Jialiang Hu, Hanmei Xu

**Affiliations:** ^1^ The Engineering Research Center of Synthetic Peptide Drug Discovery and Evaluation of Jiangsu Province, China Pharmaceutical University, Nanjing, China; ^2^ State Key Laboratory of Natural Medicines, Ministry of Education, China Pharmaceutical University, Nanjing, China

**Keywords:** cholangiocarcinoma, gemcitabine, resistance, lactylation, ITGB4

## Abstract

**Background:**

Intrahepatic cholangiocarcinoma (iCCA) is a highly aggressive malignancy of the bile ducts, and resistance to gemcitabine, a first-line chemotherapy, significantly complicates treatment. Despite extensive research, the molecular mechanisms underlying gemcitabine resistance in iCCA are not fully understood. This study aims to identify key genes associated with gemcitabine resistance in iCCA, investigate the role of lactylation, and propose potential therapeutic targets.

**Methods:**

A comprehensive bioinformatics analysis was conducted using publicly available transcriptomic data from gemcitabine-resistant iCCA cell lines and patient samples. Differential expression analysis was performed to identify upregulated and downregulated genes. GSEA were used to explore relevant molecular pathways. Immune landscape analysis was carried out using CIBERSORT to assess immune cell infiltration in the tumor microenvironment. Key resistance-related genes were identified through Lasso, RF, and SVM-REF analyses. ITGB4 function was further validated *in vitro* by siRNA knockdown in HUCCT1 and RBE cells, followed by cell viability and apoptosis assays with or without gemcitabine treatment.

**Results:**

Pathway analysis revealed the involvement of cell cycle regulation, DNA replication, and p53 signaling in gemcitabine resistance. The high group associated with resistance showed significantly worse survival outcomes, with a positive correlation between resistance and lactylation levels. Immune landscape analysis indicated altered immune cell infiltration, including increased M2 macrophages and decreased CD8^+^ T cells in the high group. Key resistance-related genes, including *CDC20*, *H2AX*, *HK2*, and *ITGB4*, were identified as critical in drug resistance. Experimentally, ITGB4 knockdown markedly enhanced gemcitabine’s antiproliferative and pro-apoptotic effects on cholangiocarcinoma cells, supporting its role in mediating resistance. Molecular docking revealed Dioscin and Deacetyllanatoside C as potential ITGB4-interacting compounds.

**Conclusion:**

This study sheds light on the molecular mechanisms of gemcitabine resistance in iCCA, emphasizing lactylation’s role and the significance of immune modulation. ITGB4 is identified as a promising therapeutic target, and the findings suggest that targeting these genes could help overcome resistance in iCCA.

## 1 Introduction

Cholangiocarcinoma is a highly aggressive and often fatal malignancy of the biliary tract, which exhibits a rising incidence globally (Ilyas and Gores, 2013; Banales et al., 2020). The disease is classified into three main subtypes based on its anatomical location: intrahepatic cholangiocarcinoma (iCCA), perihilar cholangiocarcinoma (pCCA), and distal cholangiocarcinoma (dCCA) (Razumilava and Gores, 2014; Brindley et al., 2021). CCA is often diagnosed at advanced stages, and its prognosis remains poor due to its tendency to be resistant to conventional treatments (Høgdall et al., 2018; Cantallops Vilà et al., 2024). Among the various subtypes, iCCA is particularly prevalent in Asian countries, where it is associated with high rates of hepatitis B and C viral infections (Pascale et al., 2023). In general, the incidence of CCA is further influenced by environmental and occupational risk factors, such as liver fluke infections and long-term exposure to carcinogenic chemicals (Watanapa and Watanapa, 2002; Vale et al., 2020; Brandi et al., 2022; Smout et al., 2024). Despite the challenges in diagnosing iCCA at an early stage, surgical resection remains the only potentially curative treatment, although only 20%–30% of patients are eligible for surgery (Cillo et al., 2019). Even after surgical intervention, recurrence rates remain high, and the 5-year survival rate ranges from 25% to 40% (Mazzaferro et al., 2020).

In the past decade, gemcitabine-based chemotherapy, often in combination with cisplatin, has been considered the standard first-line treatment for advanced CCA, offering modest survival benefits (Li et al., 2024). However, the overall response rate to this treatment remains low, and many patients eventually develop resistance to gemcitabine. A second-line treatment option, FOLFOX (fluorouracil, leucovorin, and oxaliplatin), has also shown limited efficacy, with only a subset of patients experiencing prolonged survival (Merters and Lamarca, 2023). The effectiveness of chemotherapy is often hampered by multiple factors, including tumor heterogeneity, a complex microenvironment, and the toxic side effects of chemotherapy drugs (Banales et al., 2020). Therefore, there is a pressing need to explore alternative therapeutic strategies, particularly those targeting molecular pathways or leveraging immunotherapy.

One of the significant challenges in CCA treatment is the development of chemoresistance, which is influenced by both the tumor microenvironment and intrinsic tumor cell mechanisms (Kobayashi et al., 2019; Biffi and Tuveson, 2021; Saw et al., 2022; Pan et al., 2023). Recent studies have highlighted lactylation as a novel epigenetic and metabolic modification contributing to tumor drug resistance. For example, histone lactylation at H4K12, catalyzed by p300, regulates gene expression to inhibit ferroptosis and promote chemoresistance in cancer stem cells, indicating a critical role of lactylation in tumor cell survival under therapeutic stress (Deng et al., 2025). Furthermore, lactylation of DNA repair proteins such as XLF at K288, modulated by the GCN5-XLF axis, enhances non-homologous end joining (NHEJ) efficiency, thereby facilitating DNA repair and contributing to chemoresistance (Jin et al., 2025). Targeting this modification presents a promising avenue for improving chemotherapy efficacy. Additionally, metabolic adaptations involving glutamine metabolism via acetylated malate enzyme 2 (ME2) promote lactate production, which in turn facilitates protein lactylation, enhancing DNA repair and resistance mechanisms (Zheng et al., 2025). This metabolic reprogramming complements the classical Warburg effect and reveals lactylation as a key link between metabolism and epigenetic regulation in cancer therapy resistance. These findings collectively underscore the emerging significance of lactylation in chemoresistance, which remains largely unexplored in iCCA and warrants further investigation.

In this study, we aim to explore the molecular mechanisms underlying gemcitabine resistance in iCCA, with a particular focus on the role of lactylation. We have collected a gene set associated with gemcitabine resistance in iCCA and conducted bioinformatics analyses to identify potential therapeutic targets. Our findings highlight several key genes and pathways that may serve as promising candidates for targeted therapy to overcome gemcitabine resistance, opening new avenues for improving treatment outcomes in iCCA patients. Additionally, we investigate the relationship between lactylation and drug resistance to identify potential biomarkers or therapeutic targets for future clinical applications.

## 2 Materials and methods

### 2.1 Data preparation

The dataset GSE116118 was downloaded from the GEO database (https://www.ncbi.nlm.nih.gov/geo/), which includes transcriptomic data from gemcitabine-resistant cholangiocarcinoma cell lines as well as control cell lines. GSE244807 comprises transcriptomic data from 246 cholangiocarcinoma tissue samples along with corresponding clinical survival information of the patients. GSE26566 contains transcriptomic data from 6 normal bile duct tissues and 104 cholangiocarcinoma samples. Additionally, GSE210067 includes single-cell transcriptomic data from 4 cholangiocarcinoma tissue samples.

### 2.2 Differentially expressed genes analysis

Differential gene expression analysis was performed using the limma package in R version 4.3.3. The control and experimental samples were selected and combined for comparison. The raw expression matrices were log2-transformed and quantile-normalized before analysis. A linear model was constructed to assess gene expression differences between the two groups, with differential expression evaluated using moderated t-statistics and *p*-values obtained from the eBayes function. Significant genes were visualized using heatmaps to display expression patterns and volcano plots to highlight genes with large fold changes and statistically significant *p*-values.

### 2.3 Univariate Cox analysis

In this study, we performed univariate Cox proportional hazards regression to evaluate the relationship between gene expression and patient survival. Survival data, including follow-up time (futime) and survival status (fustat), were used to model the risk associated with each gene. The gene expression data was preprocessed by filtering out low-expression genes (mean expression <0.5) and then transposing the dataset. For each gene, we fitted a Cox regression model to estimate the hazard ratio (HR) and its 95% confidence interval, along with the *p*-value. Genes with a *p*-value less than 0.05 were considered statistically significant.

### 2.4 Enrichment analysis

Gene Ontology (GO) and Kyoto Encyclopedia of Genes and Genomes (KEGG) pathway enrichment analyses were performed to investigate the biological functions and pathways of gene set. First, gene symbols were converted to Entrez IDs using the org.Hs.eg.db package, and genes without valid Entrez IDs were excluded. GO enrichment was carried out with the clusterProfiler package, considering all ontologies and filtering results based on *p*-value <0.05. For KEGG analysis, the enrichKEGG function was used with human pathways, and significant pathways were identified with the same filtering criteria. The results were visualized using bar plots and bubble plots to highlight the most significant GO terms and KEGG pathways.

### 2.5 Gene sets construction

The resistance-related gene set was defined as the intersection of genes upregulated in gemcitabine-resistant cholangiocarcinoma cells compared with their control cells, upregulated in cholangiocarcinoma tissues compared with normal bile duct tissues, and significantly associated with patient prognosis by univariate Cox regression. The lactylation-related gene set was obtained from GeneCards (https://www.genecards.org/) by searching the keyword “lactylation.” These gene sets were used for ssGSEA and downstream analyses.

### 2.6 ssGSEA analysis

Single-sample Gene Set Enrichment Analysis (ssGSEA) was performed to assess the activity of predefined gene sets in individual samples. The GSVA package was used to calculate ssGSEA scores for each sample across the specified gene sets. The “ssgsea” method was applied with Gaussian kernel smoothing, and the scores were ranked in absolute terms to evaluate gene set enrichment in each individual sample.

### 2.7 GSEA analysis

In this study, differential enrichment analysis between two sample groups was performed using GSEA version 4.3.2. To ensure the accuracy of the enrichment analysis, genes were ranked based on their differential expression between the groups, with the ranking criterion being the “NES” (Normalized Enrichment Score) value. The KEGG and REACTOME gene sets used in the analysis were derived from MSigDB, covering cellular functions, signaling pathways, and other biological processes. The GSEA was run with 1,000 permutations, using the “phenotype” permutation type, with the minimum and maximum gene set sizes set to 15 and 500, respectively. A *p*-value of less than 0.05 was considered statistically significant for identifying enriched pathways.

### 2.8 CIBERSORT analysis

To assess immune cell infiltration in each sample’s transcriptomic data, CIBERSORT was used to estimate the abundance of 22 immune cell types based on gene expression profiles. The LM22 immune cell signature matrix was used, and the CIBERSORT R script was run with 1,000 permutations to assess significance. Quantile normalization was also performed on the mixture data to correct for distribution differences across samples. After standardizing both the signature matrix and the mixture data, CIBERSORT applied Support Vector Regression (SVR) to estimate immune cell proportions for each sample.

### 2.9 WGCNA analysis

In this study, Weighted Gene Co-expression Network Analysis (WGCNA) was conducted to identify gene modules associated with clinical traits. First, the gene expression data was processed by selecting the top 25% of genes with the highest variance. Samples were clustered to detect outliers, and outlier samples were removed. A soft-thresholding power was determined by evaluating scale-free topology fit indices across powers ranging from 1 to 20, selecting the lowest power achieving a fit index above 0.9 to ensure approximate scale-free network topology. Using the selected soft power, an adjacency matrix was constructed and transformed into a Topological Overlap Matrix (TOM) to measure gene connectivity. Gene modules were identified by hierarchical clustering of genes based on TOM dissimilarity, followed by dynamic tree cutting with a minimum module size set to 100 genes and deepSplit parameter set to 2 to allow for moderate sensitivity in module detection. Module eigengenes (MEs) were calculated to represent each module’s expression profile. The correlations between module eigengenes and traits were computed using Pearson correlation, with *p*-values calculated to assess significance. Key genes within modules were defined as hub genes based on thresholds of module membership (MM) > 0.8 and gene significance (GS) > 0.5 for the trait of interest, highlighting genes highly connected within the module and strongly associated with phenotypes.

### 2.10 Machine learning

In this study, we utilized three machine learning techniques—LASSO regression, Random Forest, and SVM-REF—to perform feature selection on gene expression data for disease classification. First, for LASSO regression, the data was normalized, and a binary classification model was trained using cv.glmnet with 10-fold cross-validation and L1 regularization (alpha = 1). The optimal lambda value was selected, and the genes with non-zero coefficients were extracted as biomarkers. In the RF (Random Forest) analysis, the model was trained using 500 trees, and the importance of genes was assessed, with key genes visualized based on their importance scores. The optimal number of trees was determined by minimizing the error rate, and the most important genes were selected for further investigation. Lastly, we applied SVM-REF (Support Vector Machine Recursive Feature Elimination) with 10-fold cross-validation to rank genes based on their contribution to disease classification. The top-ranked genes were identified, and the model’s performance was evaluated by visualizing error rates and accuracy.

### 2.11 Nomogram analysis

In this analysis, a nomogram was developed to predict overall survival based on gene expression data. A Cox proportional hazards model was applied to survival data, using genes as predictors. The model was used to estimate survival probabilities at 1, 3, and 5 years. The nomogram was visualized to show the contribution of each gene to survival prediction. To evaluate the accuracy of the nomogram, calibration curves for 1-year, 3-year, and 5-year survival were plotted, comparing predicted survival probabilities with observed outcomes using bootstrapping.

### 2.12 Single-cell analysis

In this study, single-cell RNA sequencing data from the GSE210067 dataset were processed and analyzed using Seurat. After combining the datasets, cells were filtered based on quality control parameters such as feature count (200-7500 features), mitochondrial gene percentage (<20%), and total RNA count (>1000), resulting in a cleaned dataset for downstream analysis. The merged dataset was normalized, and variable features were identified before scaling the data. To correct for batch effects across different conditions, Harmony integration was applied. Principal component analysis (RunPCA, npcs = 50) was performed, followed by clustering of cells with FindNeighbors (dims = 1:30) and FindClusters (resolution range 0.1–1.2). The optimal clustering solution was selected based on cluster stability, visualized using UMAP for dimensionality reduction. The final cell type annotations were visualized using UMAP, with clusters labeled accordingly.

### 2.13 CellChat analysis

In this analysis, we used CellChat to explore cell-cell communication networks from single-cell RNA sequencing data. The raw count data were normalized and log-transformed, and a CellChat object was created using the expression data and cell type annotations. We employed the human receptor-ligand database (Cell-ChatDB.human) to identify significant signaling pathways, and detected over-expressed genes and ligand-receptor interactions within each cell cluster. Communication probabilities between cells were estimated, and less abundant cell types were filtered out. The communication data were analyzed and visualized through circular network plots, showing interaction number and strength, as well as individual ligand-receptor interactions.

### 2.14 Single-cell GSVA analysis

In this analysis, Gene Set Variation Analysis (GSVA) was applied to single-cell RNA sequencing data to assess pathway activity across different cell types. The analysis used three gene sets: c2.cp.kegg_legacy.v2024.1.Hs.symbols.gmt, c2.cp.pid.v2024.1.Hs.symbols.gmt, and h.all.v2024.1.Hs.symbols.gmt. The gene sets were loaded and processed using the GSVA method with raw count data from the Seurat object. GSVA scores were computed using the Poisson distribution and run in parallel for efficiency. The pathway activity scores were averaged for each cell type, and a heatmap was generated to visualize the results.

### 2.15 Molecular docking and molecular dynamics simulation

In this study, molecular docking of ITGB4 with 324 FDA-approved natural products was conducted using AutoDock Vina. The protein structure was obtained from the UniProt database (https://www.uniprot.org/) based on AlphaFold predictions, and the natural products were prepared in PDBQT format. A grid box was set to cover potential binding sites, and docking simulations were performed to evaluate binding affinities. The lowest binding energy pose was selected as the optimal binding mode, and interactions were analyzed using PyMOL.

For RMSD analysis, molecular dynamics (MD) simulations were performed using GROMACS with the AMBER99SB force field and SPCE water model. The protein was processed with pdb2gmx, and a cubic simulation box was created with editconf (box distance 1.0 nm). Solvent molecules were added using solvate, and the system was neutralized with ions through genion. Energy minimization was followed by NVT equilibration (100 ps) and NPT equilibration (100 ps) equilibration to stabilize the system, and production MD simulations were run to observe structural dynamics.

### 2.16 siRNA transfection

Human cholangiocarcinoma cell lines HUCCT1 and RBE were cultured in RPMI-1640 medium supplemented with 10% fetal bovine serum (FBS) under standard conditions (37 °C, 5% CO_2_). For siRNA transfection, cells were seeded in 6-well plates and grown to approximately 60%–70% confluency. siRNAs were synthesized by Sangon Biotech (Shanghai, China) and first diluted in jetPRIME® buffer (Polyplus-transfection SA, France), followed by vortexing for 10 s and brief centrifugation. jetPRIME® transfection reagent was then added to the diluted siRNA solution, vortexed for 1 s, briefly centrifuged, and incubated at room temperature for 10 min. The resulting transfection mixture was added dropwise to the cells in serum-containing medium. Cells were incubated for 48 h prior to subsequent experiments. The sequences of the siRNAs used in this study are listed in Supplementary Table S1.

### 2.17 RNA extraction and quantitative Real-Time PCR (qRT-PCR)

Total RNA was extracted from HUCCT1 and RBE cells using TRNzol Universal Reagent (TIANGEN, China) according to the manufacturer’s instructions. Complementary DNA (cDNA) was synthesized using the HiScript® IV RT SuperMix for qPCR (+gDNA wiper) (Vazyme, China), following the recommended protocol to eliminate genomic DNA contamination. qRT-PCR was performed using Taq Pro Universal SYBR qPCR Master Mix (Vazyme, China) on a QuantStudio Real-Time PCR system (Applied Biosystems, United States). The relative expression of target genes was calculated using the 2^−ΔΔCt^ method, and *GAPDH* was used as the internal control. All primers were synthesized by Sangon Biotech (Shanghai, China). The specific primer sequences used in this study are listed in Supplementary Table S2.

### 2.18 Western blot

Total cellular proteins were extracted using RIPA lysis buffer supplemented with protease inhibitors. Protein concentrations were determined using the BCA method. Equal amounts of protein were separated by SDS-PAGE and transferred onto PVDF membranes. After blocking with 5% non-fat milk in TBST for 1 h at room temperature, the membranes were incubated overnight at 4 °C with rabbit monoclonal antibodies against ITGB4 and GAPDH (both from HUABIO, China). After washing with TBST, the membranes were incubated with HRP-conjugated goat anti-rabbit IgG secondary antibody (HUABIO, China) for 1 h at room temperature. Protein bands were visualized using enhanced chemiluminescence (ECL) reagent (Abbkine, China) and detected using a chemiluminescence imaging system.

### 2.19 Cell viability assay

Cell viability was evaluated using the Cell Counting Kit-8 (CCK-8; YEASEN, China) in accordance with the manufacturer’s instructions. HUCCT1 and RBE cells were seeded in 96-well plates at a density of 5 × 10^3^ cells per well and allowed to adhere overnight. Cells were subjected to either siRNA transfection or gemcitabine (Tokyo Chemical Industry, Japan) treatment for 48 h. Subsequently, 10 μL of CCK-8 solution was added to each well, followed by incubation at 37 °C for 1–2 h. The absorbance at 450 nm was measured using a microplate reader to determine cell viability.

### 2.20 Apoptosis assay

Cell apoptosis was evaluated using the Annexin V-APC/PI Apoptosis Detection Kit (MULTI SCIENCES, China) following the manufacturer’s protocol. Briefly, cells were harvested, washed twice with cold PBS, and resuspended in 1× binding buffer at a concentration of 1 × 10^6^ cells/mL. Subsequently, 100 μL of the cell suspension was incubated with 5 μL of Annexin V-APC and 10 μL of propidium iodide (PI) for 10–15 min at room temperature in the dark. After incubation, 400 μL of binding buffer was added, and apoptotic cells were analyzed by flow cytometry.

### 2.21 Statistical analysis

All statistical analyses were performed using GraphPad Prism 9. Data are presented as the mean ± standard deviation (SD) from at least three independent experiments. Statistical significance between two groups was assessed using an unpaired two-tailed Student’s t-test. A *p*-value <0.05 was considered statistically significant.

## 3 Results

### 3.1 Identification of 36 upregulated genes associated with gemcitabine resistance and poor prognosis in cholangiocarcinoma

**FIGURE 1:**
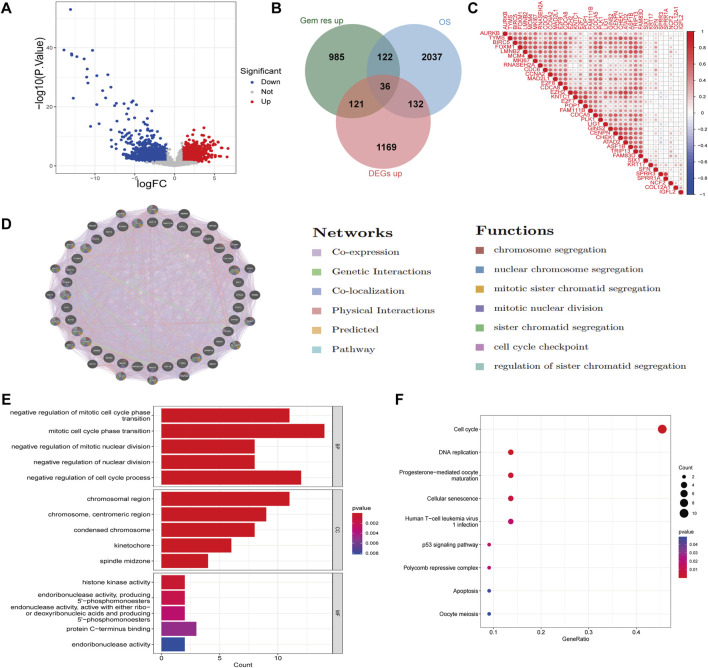
Identification of gemcitabine resistance-related DEGs in cholangiocarcinoma. **(A)** Vol-cano plot showing DEGs in cholangiocarcinoma tissues compared to normal bile duct tissues. Red dots represent upregulated genes and blue dots represent downregulated genes, using a threshold of |logFC| > 1 and *p*-value <0.05. **(B)** Venn diagram illustrating the intersection of upregulated genes in gemcitabine-resistant cholan-giocarcinoma cell lines, risk-related genes from univariate Cox regression analysis, and upregu-lated genes in cholangiocarcinoma tissues, identifying 36 common genes. **(C)** Correlation heatmap of the 36 overlapping genes. **(D)** PPI network analysis of the 36 genes using the GeneMANIA database. Nodes represent genes, and edges represent predicted or known functional interactions, including physical interactions, co-expression, and pathway co-membership. **(E)** GO enrichment analysis of the 36 genes, showing significant en-richment in biological processes, cellular components, and molecular functions. **(F)** KEGG pathway analysis of the 36 genes.

In this study, we performed Differentially Expressed Genes (DEGs) analysis on the GSE116118 dataset, which includes gemcitabine-resistant cholangiocarcinoma cell line MT-CHC01 and a control cell line. Using a threshold of |logFC| > 1 and *p*-value <0.05, we identified 1,264 upregulated and 1,238 downregulated genes in the resistant group compared to the control group (Supplementary Figure S1A). Furthermore, we analyzed the GSE26566 dataset, which compares cholangiocarcinoma tissues to normal bile duct tissues. Applying the same threshold, we found 1,458 upregulated and 1,693 downregulated genes in cholangiocarcinoma tissues (Figure 1A; Supplementary Figure S1B). Next, we conducted univariate Cox regression analysis on the GSE244807 dataset, which includes cholangiocarcinoma patients with clinical survival data. Genes with a *p*-value less than 0.05 were considered risk-related genes, yielding a total of 2,327 genes. We then intersected the upregulated genes from the resistant group, risk-related genes, and the upregulated genes in cholangiocarcinoma tissues, resulting in 36 common genes (Figure 1B). Correlation analysis of these 36 genes revealed that most of them exhibited positive correlations, suggesting a regulatory relationship among them (Figure 1C). Protein-protein interaction (PPI) network analysis using the GeneMANIA database further revealed that these genes are primarily involved in co-expression, genetic interactions, and co-localization. They are predominantly associated with biological pro-cesses such as chromosome segregation, mitotic sister chromatid segregation, and cell cycle checkpoint regulation (Figure 1D). We also performed GO and KEGG enrichment analyses on the 36 genes (Figures 1E,F). GO enrichment analysis showed that the biological processes (BP) these genes are mainly involved in include the negative regulation of mitotic cell cycle phase transition, mitotic cell cycle phase transition, and negative regulation of nuclear division. In terms of cellular components (CC), the genes are primarily located in the chromosomal region, centromeric region, and condensed chromosome. For molecular function (MF), the genes are mainly associated with histone kinase activity. KEGG pathway analysis revealed that these genes are mainly involved in pathways such as the cell cycle, DNA replication, and the p53 signaling pathway. Clinically, this suggests that gemcitabine resistance in CCA may be linked to heightened proliferative signaling and defective checkpoint regulation, which could potentially be exploited for targeted therapy or predictive biomarker development.

### 3.2 Gemcitabine-resistant CCA subtypes exhibit poor prognosis, elevated lactylation, and enrichment in cell cycle pathways

**FIGURE 2:**
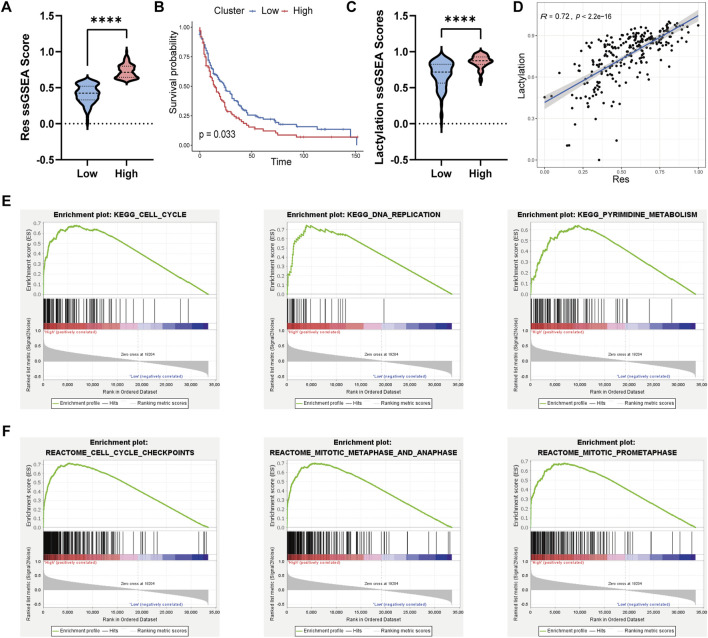
Molecular signaling pathway differences between different cholangiocarcinoma sub-types. **(A)** Violin plot comparing the ssGSEA scores of the drug resistance-related gene set be-tween the high and low groups in cholangiocarcinoma patients. **(B)** Kaplan-Meier survival curve illustrating the significantly lower survival rates of patients in the high group compared to the low group. **(C)** Violin plot showing that the high group had significantly higher lactylation-related ssGSEA scores compared to the low group. **(D)** Spearman correlation analysis between drug re-sistance-related ssGSEA scores and lactylation scores. **(E)** GSEA enrichment analysis using the KEGG gene set, showing significant pathways in the high group. **(F)** GSEA enrichment analysis using the Reactome gene set, highlighting pathways enriched in the high group.

Based on the 36 genes identified, we established a drug resistance-related gene set. We then performed ssGSEA on the transcriptomic data of 246 cholangiocarcinoma patients from the GSE244807 dataset using this resistance-related gene set. Patients were divided into high and low groups based on their ssGSEA scores, using a threshold of 0.6 to define the groups. As shown in Figure 2A, the high group exhibited significantly higher ssGSEA scores for the resistance-related gene set compared to the low group. Further survival analysis using clinical data demonstrated that patients in the high group had significantly lower survival rates than those in the low group, as illustrated in Figure 2B. To explore additional molecular characteristics, we performed ssGSEA analysis on a lactylation-related gene set. The results revealed that the high group had significantly higher lactylation levels compared to the low group (Figure 2C). Furthermore, Spearman correlation analysis showed a significant positive correlation between the ssGSEA scores of the drug resistance-related gene set and lactylation scores (Figure 2D). To validate the robustness of these findings, we applied the same ssGSEA analysis to the validation dataset, GSE26566, which includes 104 cholangiocarcinoma samples. Consistent with the initial results, the high group in the validation cohort also displayed significantly higher drug resistance scores compared to the low group (Supplementary Figure S2A). Moreover, analysis of lactylation scores between the two groups in the validation dataset revealed that the high group had significantly higher lactylation levels, and these scores were positively correlated with the drug resistance scores (Supplementary Figure S2B,C). To further explore the underlying mechanisms, we performed GSEA to identify pathway differences between the high and low groups. GSEA revealed significant enrichment of pathways related to Cell Cycle, DNA Replication, and Pyrimidine Metabolism in the high group (Figure 2E). Additionally, Reactome pathway analysis identified Cell Cycle Checkpoints, Mitotic Metaphase and Anaphase, and Mitotic Prometaphase as significantly enriched in the high group (Figure 2F). The molecular subtype may represent a high-risk patient group that could benefit from therapies targeting lactylation-associated repair mechanisms or cell cycle checkpoints, in combination with gemcitabine.

### 3.3 Immune-evasive phenotype characterizes the gemcitabine-resistant iCCA subtype

**FIGURE 3:**
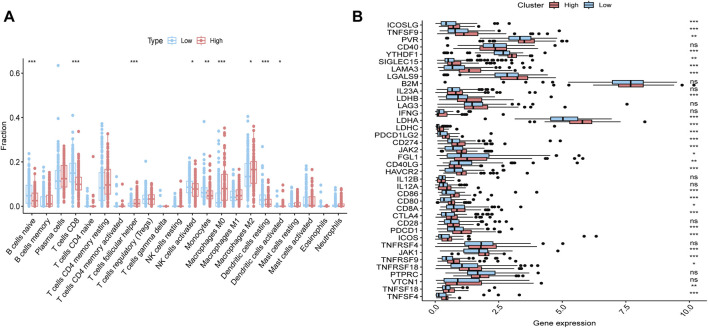
Immune landscape differences between cholangiocarcinoma subtypes. **(A)** CIBERSORT analysis comparing the infiltration levels of 22 immune cell types between the high and low groups. **(B)** Differential expression of immune checkpoint genes between the high and low groups.

To further investigate the immune characteristics between the groups with high and low ssGSEA scores, we performed CIBERSORT analysis on the transcriptomic data of each sample, which allowed us to assess the infiltration levels of 22 different immune cell types within the tumor microenvironment. The results showed that, in the high group, the infiltration levels of Plasma cells, CD4 memory resting T cells, and M2 macrophages were significantly higher compared to the low group, whereas the infiltration levels of CD8 T cells, activated NK cells, and resting dendritic cells were notably lower in the high group (Figure 3A). Furthermore, we conducted differential expression analysis of immune checkpoint genes between the two groups. The results revealed that many immune checkpoint genes, including CTLA4, ICOSLG, and LAMA3, were significantly upregulated in the high group compared to the low group (Figure 3B). These findings suggest that the high group may exhibit an altered immune landscape, potentially contributing to the tumor’s resistance to therapy and its ability to evade immune surveillance. Immune-evasive phenotype implies that gemcitabine-resistant iCCA patients might also benefit from combination strategies involving immune checkpoint blockade, particularly in cases where lactylation and cell cycle dysregulation co-occur.

### 3.4 Differentially expressed genes and WGCNA analysis results in different iCCA subtypes

**FIGURE 4:**
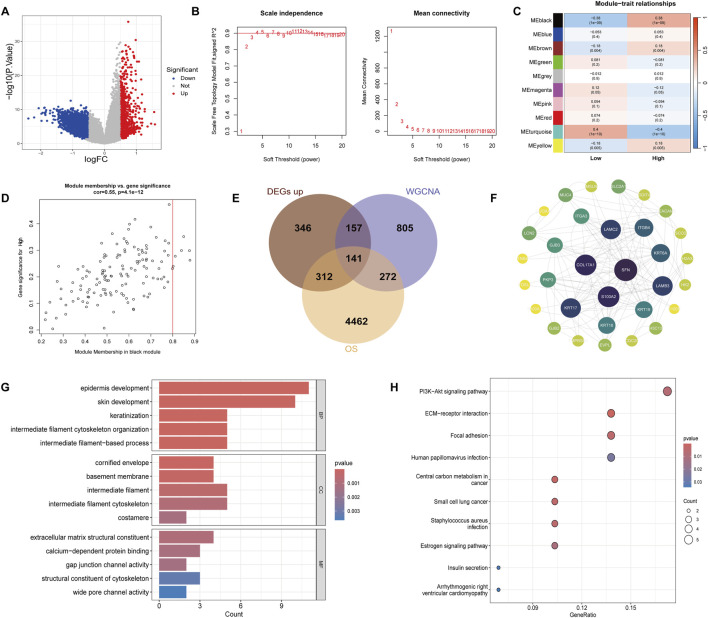
Identification of DEGs and WGCNA results. **(A)** Volcano plot of DEGs between the high and low groups. Red dots represent upregulated genes and blue dots represent downregulated genes, using a threshold of |logFC| > 0.5 and *p*-value <0.05. **(B)** Soft-thresholding power analysis for WGCNA, with a threshold of 3 selected for scale-free topology. **(C)** Correlation heatmap between identified gene modules and high/low gene scores. **(D)** Scatter plot depicting the correlation between gene module member-ship and gene importance in the black module. **(E)** Venn diagram showing the intersection of upregulated genes in the high group, black module genes from WGCNA, and risk-associated genes, identifying 141 common genes. **(F)** PPI network of the 141 intersecting genes constructed using the STRING database (confidence score >0.4) and visualized in Cytoscape, highlighting SFN, LAMC2, and ITGB4 as key nodes. **(G)** GO enrichment analysis of the 141 genes, showing involvement in biological processes, cellular components, and molecular functions. **(H)** KEGG enrichment analysis showing the key pathways these genes are involved in.

To further explore the molecular differences between the high and low groups, we performed differential gene expression analysis and WGCNA. Using a threshold of |logFC| > 0.5 and a *p*-value <0.05, a total of 3432 DEGs were identified, with 956 genes upregulated and 2476 genes downregulated in the high group (Figure 4A). The optimal soft threshold for constructing the gene co-expression network was determined to be 3 based on the scale-free topology fitting index (R2) (Figure 4B). Using average linkage hierarchical clustering and the soft threshold power, we identified distinct gene modules The correlation between the gene modules and the high/low gene scores was visualized in a correlation plot, showing that the black module had a strong positive correlation with the high group (correlation = 0.38, *p*-value = 1 × 10^−9^), and this module was selected for subsequent analysis (Figure 4C). Additionally, the correlation between the black module and gene importance is shown in Figure 4D. We further intersected the upregulated genes in the high group, the black module genes, and the risk-associated genes, resulting in a list of 141 intersecting genes (Figure 4E). The PPI analysis was conducted for these 141 genes using the STRING database, excluding proteins without interactions. The PPI network was visualized using Cytoscape, highlighting the central proteins SFN, LAMC2, and ITGB4, which were identified as key nodes within the network (Figure 4F). To gain further insights into the biological functions and signaling pathways involved, we performed GO and KEGG enrichment analyses. The GO analysis revealed that the BP associated with these genes were primarily related to intermediate filament cytoskeleton organization and intermediate filament-based processes. CC analysis showed localization to intermediate filaments and intermediate filament cytoskeleton, while MF analysis identified key roles in extracellular matrix structural constituent and gap junction channel activity (Figure 4G). The KEGG enrichment analysis revealed that these genes were mainly involved in key signaling pathways, including the PI3K-Akt signaling pathway, ECM-receptor interaction, and focal adhesion (Figure 4H). These results suggest that the 141 intersecting genes, particularly those involved in intermediate filament organization and ECM interactions, may play significant roles in the drug resistance mechanisms of cholangiocarcinoma. The identified key proteins, such as SFN, LAMC2, and ITGB4, along with their associated pathways, could serve as potential biomarkers or therapeutic targets for overcoming resistance in this cancer type.

### 3.5 *CDC20*, *H2AX*, *HK2*, *H3C13*, and *ITGB4* identified as Robust predictors of resistance and prognosis in iCCA

**FIGURE 5:**
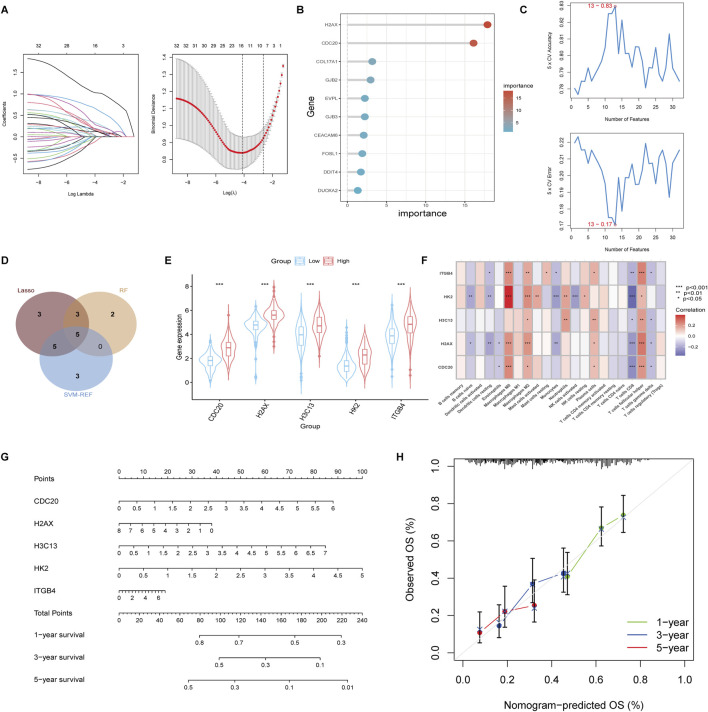
Identification of feature genes using Lasso, Random Forest (RF), and SVM-REF analysis. **(A)** Lasso regression analysis identified 16 genes with non-zero coefficientse. **(B)** Random Forest analysis ranked the importance of the 32 core genes and selected the top 10 most significant genes. **(C)** SVM-REF analysis determined the optimal model threshold at 13 genes, achieving the lowest error rate and highest accuracy. **(D)** Venn diagram displaying the intersection of genes identified by Lasso, RF, and SVM-REF analyses, revealing five key feature genes: *CDC20*, *H2AX*, *H3C13*, *HK2*, and *ITGB4*. **(E)** Boxplot comparing the expression levels of the five feature genes between the high and low groups. **(F)** Spearman correlation analysis between the expression of the five feature genes and immune cell infiltration levels. **(G)** A nomogram constructed based on the five feature genes for predicting patient prognosis. **(H)** Calibration curves for 1-, 3-, and 5-year sur-vival predictions.

To further identify potential therapeutic targets among the drug resistance-related genes in the high group, we conducted Lasso, Random Forest (RF), and SVM-REF analyses on the 32 core genes obtained from the PPI network. The Lasso analysis identified 16 genes with non-zero coefficients (Figure 5A). In the RF analysis, the importance of each gene was assessed, and the top 10 most important genes were selected for further analysis (Figure 5B). The SVM-REF analysis showed that the model achieved the lowest error rate and highest accuracy at a threshold of 13, yielding 13 key features (Figure 5C). We intersected the genes identified from all three analyses, resulting in a final list of 5 feature genes: *CDC20*, *H2AX*, *H3C13*, *HK2*, and *ITGB4* (Figure 5D). These genes were found to be significantly more highly expressed in the high group compared to the low group (Figure 5E). To assess the potential relationship between these 5 feature genes and immune cell infiltration, we performed Spearman correlation analysis between the expression levels of these genes and the infiltration levels of various immune cell types. The analysis revealed that these genes were positively correlated with Macrophages M0, Macrophages M2, and T cells follicular helper, while they showed a negative correlation with Monocytes and T cells CD8 (Figure 5F). Further survival analysis was conducted using clinical survival data along with the expression of these 5 genes. A nomogram based on these genes was constructed to predict patient prognosis (Figure 5G), and the calibration curves for 1, 3, and 5 years were plotted, showing good prediction accuracy (Figure 5H). Additionally, we analyzed the correlation between the expression of these genes and lactylation scores, revealing a positive correlation between the expression levels of all five genes and Lactylation levels (Supplementary Figure S3A). Survival analysis of these genes also indicated that high expression of these genes was associated with poor prognosis in patients (Supplementary Figure S3B). These findings suggest that the identified genes, particularly *CDC20*, *H2AX*, *H3C13*, *HK2*, and *ITGB4*, may serve as potential therapeutic targets for overcoming drug resistance in cholangiocarcinoma.

### 3.6 Epithelial subpopulations enriched in ITGB4 and lactylation signatures drive drug resistance in iCCA

**FIGURE 6:**
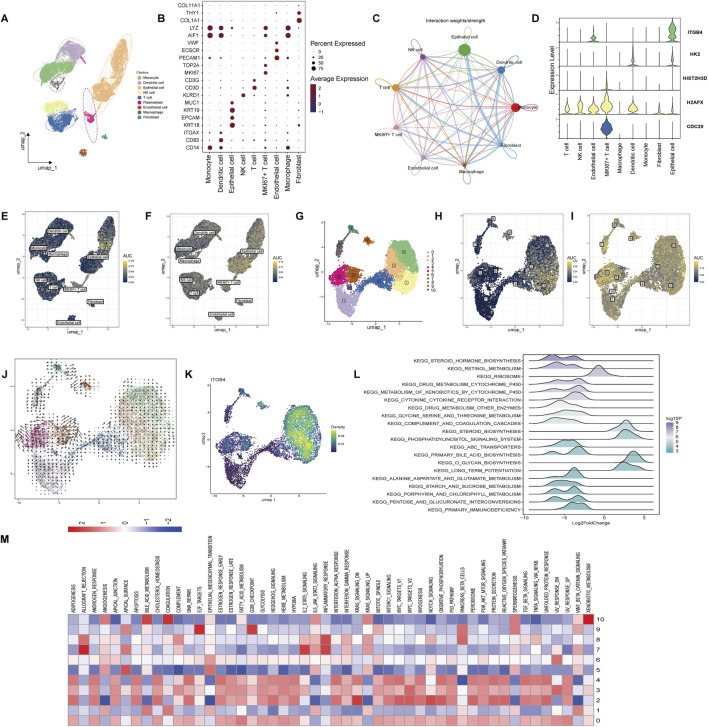
Single-cell RNA sequencing analysis of cholangiocarcinoma cell populations. **(A)** UMAP plot illustrating the clustering of major cell populations in cholangiocarcinoma tissue, in-cluding Monocytes, Dendritic cells, Epithelial cells, NK cells, T cells, MKI67 + T cells, Endothelial cells, Macrophages, and Fibroblasts. **(B)** Bubble chart displaying the expression of marker genes used to define each cell type. **(C)** Cell-cell communication network inferred using the CellChat. **(D)** Violin plot showing the expression levels of the five identified feature genes across different cell populations. **(E)** AUC-based scoring of the drug-resistance-related gene set across different subpopulations. **(F)** AUC-based scoring of the lactylation-related gene set. **(G)** UMAP plot of ep-ithelial cell subpopulations, identifying 11 distinct clusters. **(H)** AUC scores showing that the drug-resistance-related gene set is mainly enriched in subpopulations 0, 2, and 3. **(I)** AUC scores indicating that the lactylation-related gene set is significantly enriched across all epithelial sub-populations. **(J)** RNA velocity analysis indicating differentiation trajectories of epithelial cell subpopulations, with differentiation directed toward subpopulations 0, 2, and 3. **(K)** UMAP plot showing that *ITGB4* expression is predominantly enriched in subpopulations 0, 2, and 3. **(L)** GSEA pathway enrichment analysis comparing high and low ITGB4 expression groups, visual-ized in a mountain plot. **(M)** Pathway enrichment analysis of epithelial subpopulations using HALLMARK gene sets.

We analyzed the distribution of genes across various subpopulations in cholangiocarcinoma using single-cell RNA sequencing data from the GSE210067 dataset. Dimensionality reduction and clustering were performed, and cell populations were identified based on their marker genes. The cells in the cholangiocarcinoma tissue were primarily classified into the following subpopulations: Monocytes, Dendritic cells, Epithelial cells, NK cells, T cells, MKI67 + T cells, Endothelial cells, Macrophages, and Fibroblasts. These subpopulations were visualized in a UMAP plot (Figure 6A). Marker genes for each cell type were displayed in a bubble chart (Figure 6B). To assess the in-teractions between these cell types, we used the CellChat tool to analyze cell-to-cell communication. The results, shown in Figure 6C, revealed significant interactions be-tween fibroblasts and other cell types. In Supplementary Figure S4A, we further explored the receptor-ligand relationships between fibroblasts and various cell types. We then examined the distribution of the five potential therapeutic targets in each cell type. Of note, *ITGB4* was found to be enriched in epithelial cells, while *CDC20* showed a higher expression in MKI67 + T cells (Figure 6D). Additionally, we scored the drug-resistance-related gene set and lactylation-related gene set using the AUC method in different subpopulations. The drug-resistance-related gene set was primarily enriched in epithelial cells (Figure 6E), and similarly, the lactylation-related gene set also exhibited high enrichment in epithelial cells (Figure 6F). Next, we performed dimensionality reduction and clustering analysis on epithelial cells, identifying 11 distinct epithelial subpopulations (Figure 6G). AUC scores for the drug-resistance-related gene set and lactylation-related gene set were calculated for each subpopulation. The results showed that the drug-resistance-related gene set was predominantly enriched in sub-populations 0, 2, and 3 (Figure 6H), while the lactylation-related gene set was significantly enriched across all epithelial subpopulations (Figure 6I). We further analyzed the RNA velocity of epithelial cell subpopulations to infer their differentiation trajectories. The analysis indicated that the subpopulations mainly differentiated toward subpopulation 0, 2, and 3 (Figure 6J). Visualizing the expression of *ITGB4* across these subpopulations revealed that *ITGB4* was particularly enriched in subpopulations 0, 2, and 3 (Figure 6K). We performed GSEA to explore the pathways enriched in high and low *ITGB4* expression groups. The GSEA results were visualized in a mountain plot, high-lighting significant pathway enrichment (Figure 6L). Finally, to investigate the pathway activity in different epithelial cell subpopulations, we conducted pathway enrichment analysis using the KEGG, PID, and HALLMARK gene sets for each subpopulation. Subpopulations 0, 2, and 3 were significantly enriched in pathways related to DNA Repair, P53 Pathway, FAK Pathway, and VEGF-VEGFR Pathway (Figure 6M; Supplementary Figure S5). These findings suggest that *ITGB4* plays a key role in the epithelial subpopulations, particularly in those subpopulations associated with drug resistance and lactylation. The identified signaling pathways provide valuable insights into potential therapeutic targets and the molecular mechanisms driving cholangiocarcinoma progression.

### 3.7 ITGB4 knockdown reduces proliferation and increases gemcitabine sensitivity in CCA cell lines

**FIGURE 7:**
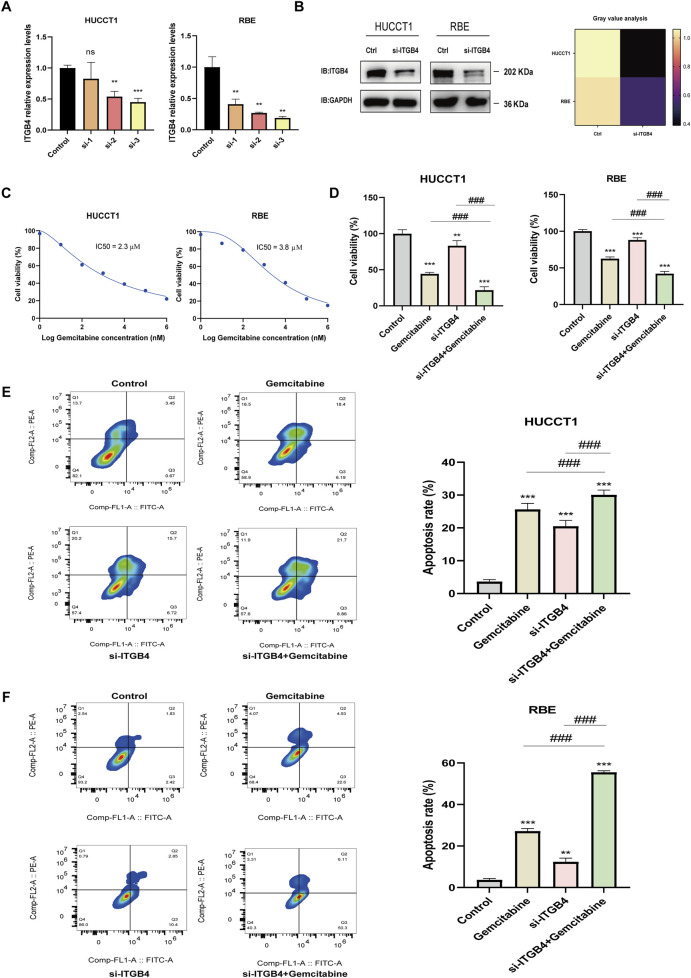
ITGB4 knockdown suppresses proliferation and enhances gemcitabine sensitivity in cholangiocarcinoma cells. **(A)** qRT-PCR analysis of *ITGB4* mRNA levels in HUCCT1 and RBE cells transfected with *ITGB4*-specific siRNAs or negative control siRNA. **(B)** Western blot analysis of ITGB4 protein expression in HUCCT1 and RBE cells after transfection with the most effective *ITGB4* siRNA or NC siRNA. GAPDH served as the loading control. **(C)** Determination of gemcitabine IC_50_ values in HUCCT1 and RBE cells by CCK-8 assay following 48 h treatment with a range of gemcitabine concentrations. **(D)** Cell viability of HUCCT1 and RBE cells transfected with *ITGB4* siRNA or NC siRNA, with or without gemcitabine treatment for 48 h, as determined by CCK-8 assay. **(E,F)** Flow cytometric analysis of apoptosis in HUCCT1 **(E)** and RBE **(F)** cells subjected to ITGB4 knockdown and/or gemcitabine treatment for 48 h using Annexin V-APC/PI staining. Data represent mean ± SD from at least three independent experiments; *p* > 0.05. ns, no significance; **, *p* < 0.01; ***, ###, *p* < 0.001.

To validate the potential involvement of ITGB4 in regulating gemcitabine sensitivity in cholangiocarcinoma cells, qRT-PCR analysis was first performed, revealing that transfection with *ITGB4*-targeting siRNA significantly reduced *ITGB4* mRNA expression in both HUCCT1 and RBE cells (Figure 7A). The siRNA exhibiting the highest knockdown efficiency was selected for subsequent experiments. Consistently, Western blot analysis confirmed a marked reduction in ITGB4 protein levels in both cell lines following siRNA transfection (Figure 7B). To determine the appropriate concentration of gemcitabine for subsequent assays, dose–response experiments were performed to calculate the half-maximal inhibitory concentration (IC_50_). The IC_50_ values were 2.3 μM for HUCCT1 cells and 3.8 μM for RBE cells (Figure 7C), and 3.8 μM was used in the following experiments. CCK-8 assays demonstrated that either ITGB4 knockdown or gemcitabine treatment alone significantly reduced cell viability in both HUCCT1 and RBE cells (Figure 7D). Notably, the combination of ITGB4 silencing and gemcitabine treatment led to a significantly greater reduction in viability compared with either treatment alone (Figure 7D). Flow cytometric analysis of apoptosis revealed that both ITGB4 knockdown and gemcitabine treatment alone significantly increased apoptosis rates in HUCCT1 and RBE cells, whereas the combination of ITGB4 knockdown with gemcitabine resulted in a further and significant increase in apoptosis compared with either single treatment (Figures 7E,F). Collectively, these results indicate that ITGB4 may play a critical role in regulating gemcitabine sensitivity in cholangiocarcinoma cells, and its silencing enhances the antiproliferative and pro-apoptotic effects of gemcitabine.

### 3.8 Molecular docking and molecular dynamics simulation results

**FIGURE 8:**
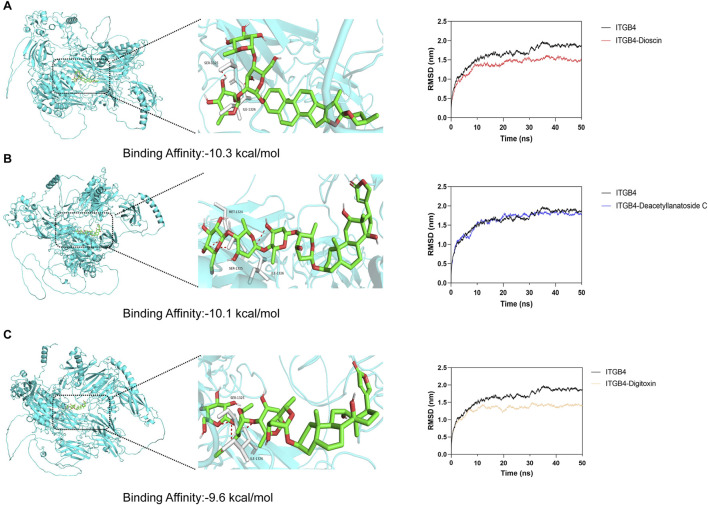
Molecular docking and molecular dynamics simulation results for ITGB4-targeting compounds. **(A–C)** Molecular docking and molecular dynamics simulation results for Dioscin, Deacetyllanatoside C, and Digitoxin.

To identify potential therapeutic compounds targeting the ITGB4 receptor, we performed molecular docking and molecular dynamics (MD) simulations. A total of 324 FDA-approved natural products were selected as candidate molecules for docking studies. The docking was conducted using AutoDock Vina, and the compounds were ranked according to their binding affinity. Among the compounds tested, Dioscin, Deacetyllanatoside C, and Digitoxin exhibited relatively lower binding affinities to ITGB4, with binding energies of −10.3 kcal/mol, −10.1 kcal/mol, and −9.6 kcal/mol, re-spectively (Figures 8A–C). To further analyze the molecular interactions, we used PyMOL to visualize the binding interactions between the small molecules and the ITGB4 receptor. Hydrogen bonds were identified between the compounds and key residues at the MET1324, SER1325, and ILE1326 positions of the receptor. To assess the stability of the receptor-ligand complexes, we performed 50 ns molecular dynamics simulations. The Root Mean Square Deviation (RMSD) of the complexes with ITGB4 was calculated. The results showed that the RMSD values of the complexes formed by Dioscin and Digitoxin with ITGB4 were lower than that of the free ITGB4 protein (Figures 8A–C). This suggests that the binding of these two compounds to ITGB4 results in more stable complexes, indicating their potential as promising candidates for further therapeutic exploration.

## 4 Discussion

iCCA remains a highly aggressive and poorly responsive malignancy with limited treatment options, particularly in its advanced stages (Rodrigues et al., 2021; Sarcognato et al., 2021). Surgical resection, the only potentially curative treatment, is only feasible in a minority of patients, with recurrence rates remaining high even after successful surgery (Gorji and Beal, 2022; Ivey et al., 2023; Ohaegbulam et al., 2023; Sahu et al., 2023). Gemcitabine, often combined with cisplatin, has been the cornerstone of systemic chemotherapy for advanced iCCA; however, the clinical efficacy of this regimen is limited by the development of resistance, leading to poor overall survival (Raggi et al., 2022). The mechanisms behind chemoresistance in iCCA are multifactorial, involving alterations in metabolism, DNA repair pathways, and the tumor microenvironment (Lin et al., 2022; Chen et al., 2023; Gao et al., 2023; Sanchon-Sanchez et al., 2023; Nagaoka et al., 2024). This chemoresistance translates into treatment failure and disease progression, underscoring the urgent need for novel biomarkers that can predict response and guide therapeutic decisions. Thus, identifying novel molecular markers and therapeutic targets to overcome gemcitabine resistance is a critical research area for improving outcomes in iCCA patients.

In this study, we identified 36 genes that are commonly upregulated in gemcitabine-resistant iCCA cell lines and clinical cholangiocarcinoma tissues. Through comprehensive bioinformatics analyses, including Cox regression, PPI networks, and gene enrichment analyses, we found that these genes were involved in critical cellular processes such as mitotic cell cycle regulation, DNA replication, and chromosomal segregation. These findings are consistent with the known molecular characteristics of iCCA, where the dysregulation of cell cycle checkpoints and DNA repair mechanisms are frequently implicated in chemotherapy resistance (Huang et al., 2022; Liu et al., 2024; Mancarella et al., 2024). Moreover, the observed enrichment of these genes in pathways like the p53 signaling pathway further highlights the complex interplay between genetic alterations and the failure of conventional chemotherapy. These results underscore the importance of cell cycle regulation as a potential therapeutic target for overcoming gemcitabine resistance in iCCA.

In addition to cell cycle regulation, our analysis of lactylation-related gene sets revealed a significant correlation between lactylation levels and drug resistance in iCCA. Lactylation, a recently identified post-translational modification, has been shown to play a role in regulating gene expression and cellular metabolism (Zhang et al., 2019; Chen et al., 2022; Wang et al., 2022; Lv et al., 2023). Our findings suggest that lactylation may contribute to the drug resistance phenotype in iCCA, possibly by modulating the expression of genes involved in metabolism and cell survival. This represents a novel mechanism by which iCCA cells may evade chemotherapeutic effects, underscoring the need for further research into lactylation’s role in cancer biology. Targeting lactylation-related pathways could complement current therapies by sensitizing resistant tumors to gemcitabine. Additionally, lactylation-associated markers hold potential as predictive biomarkers to stratify patients who may benefit from such treatments.

The immune landscape of CCA also plays a crucial role in the development of chemoresistance (Cadamuro et al., 2022). Our analysis of immune cell infiltration using CIBERSORT revealed distinct immune profiles between high and low groups. Notably, the high group exhibited higher levels of M2 macrophages, which are known to promote tumor progression and immune evasion (Cervantes-Villagrana et al., 2020; Yin et al., 2022). In contrast, key immune cell populations such as CD8^+^ T cells and activated NK cells were depleted in the high group, indicating a potential immune suppression mechanism that could facilitate tumor resistance to therapy. Furthermore, the upregulation of immune checkpoint genes, such as CTLA4 and ICOSLG, in the high group suggests that immune evasion mechanisms may be contributing to resistance. Studies have shown that anti-CTLA4 antibodies can reverse drug resistance in intrahepatic cholangiocarcinoma (Lu et al., 2024). These findings highlight the potential for combining immunotherapies with chemotherapy to overcome resistance, tailoring treatments based on immune profiling could improve clinical outcomes. Moreover, therapeutic strategies aiming to reprogram the tumor immune microenvironment, such as macrophage polarization inhibitors or immune checkpoint blockers, warrant further clinical investigation in iCCA.

While our study provides valuable insights into the molecular mechanisms underlying gemcitabine resistance in iCCA, several important limitations should be acknowledged. First, although we have complemented the bioinformatics findings with *in vitro* experiments validating the role of ITGB4 in modulating gemcitabine sensitivity, the potential involvement of lactylation remains unverified and warrants further exploration in both cell-based and *in vivo* models. Second, given the marked heterogeneity of CCA, future work should aim to identify subtype-specific biomarkers and therapeutic strategies to enable more personalized treatment. Third, although gemcitabine in combination with cisplatin is the current standard first-line regimen, our study deliberately focused on gemcitabine monoresistance to minimize confounding from additional agents and to dissect single-drug resistance mechanisms; however, resistance to combination therapy remains an important topic for future research. Importantly, ITGB4 has been implicated in modulating cell adhesion through PI3K/AKT signaling and has also been associated with drug resistance in several malignancies, and our findings suggest that targeting ITGB4 may represent a promising strategy to overcome gemcitabine resistance in iCCA, with potential clinical translation in the context of ongoing drug development for CCA (Shi et al., 2022; Fang et al., 2023; Zhu et al., 2025). Overall, our study provides a foundation for further investigation into the molecular mechanisms of gemcitabine resistance in iCCA and offers a rationale for incorporating lactylation pathway inhibitors, immune checkpoint blockade, or their combination with gemcitabine into future clinical trial designs to improve patient survival.

## 5 Conclusion

In conclusion, this study provides a comprehensive analysis of the molecular mechanisms underlying gemcitabine resistance in iCCA. We identified a set of drug resistance-related genes significantly upregulated in both gemcitabine-resistant cell lines and clinical iCCA tissues, involved in key processes like cell cycle regulation, DNA replication, and chromosomal segregation. Additionally, lactylation emerged as a novel and important modulator of drug resistance in iCCA, highlighting its potential as a biomarker and therapeutic strategy. The altered immune landscape in high groups, with changes in immune cell infiltration and upregulated immune checkpoint genes, suggests immune evasion contributes to resistance. The identification of therapeutic targets such as CDC20, H2AX, and ITGB4, combined with immune modulation and post-translational modifications, offers new avenues for overcoming resistance. Clinically, these findings underscore the potential for targeting lactylation pathways to enhance gemcitabine efficacy and overcome resistance. Future research should focus on validating lactylation’s mechanistic role in chemoresistance *in vivo* and exploring lactylation-targeted therapies to improve patient outcomes in iCCA.

## Data Availability

The original contributions presented in the study are included in the article/Supplementary Material, further inquiries can be directed to the corresponding author.
