# Supplementary material for: Integrated transcriptomic analysis identifies lactylation-linked gemcitabine resistance and therapeutic targets in intrahepatic cholangiocarcinoma
Source: Front Cell Dev Biol. 2025 Sep 1;13:1611434. doi: 10.3389/fcell.2025.1611434 (PMC12433945; doi:10.3389/fcell.2025.1611434)
Supplement: Supplementary file 1 [file DataSheet1.docx]

Supplementary Material

**Supplementary Table 1. Sequences of siRNAs used in this study.**

| **siRNA Name** | **Sense** | **Antisense** |
| --- | --- | --- |
| **si-*ITGB4*-1** | GCGCCUACUGCACAGACGA/dT//dT/ | UCGUCUGUGCAGUAGGCGC/dT//dT/ |
| **si-*ITGB4*-2** | CAGACGAGAUGUUCAGGGA/dT//dT/ | UCCCUGAACAUCUCGUCUG/dT//dT/ |
| **si-*ITGB4*-3** | CAGCGACUACACUAUUGGA/dT//dT/ | UCCAAUAGUGUAGUCGCUG/dT//dT/ |

**Supplementary Table 2. Primer sequences for qRT-PCR analysis.**

| **Primer Name** | **Sequence (5'→3')** |
| --- | --- |
| ***ITGB4*-F** | GCCTGAGGATGACGACGAGAAG |
| ***ITGB4*-R** | CTGGCTTGCTCCTTGATGATGGT |
| ***GAPDH*-F** | TGGACCTGACCTGCCGTCTA |
| ***GAPDH*-R** | AGTGGGTGTCGCTGTTGAAGT |


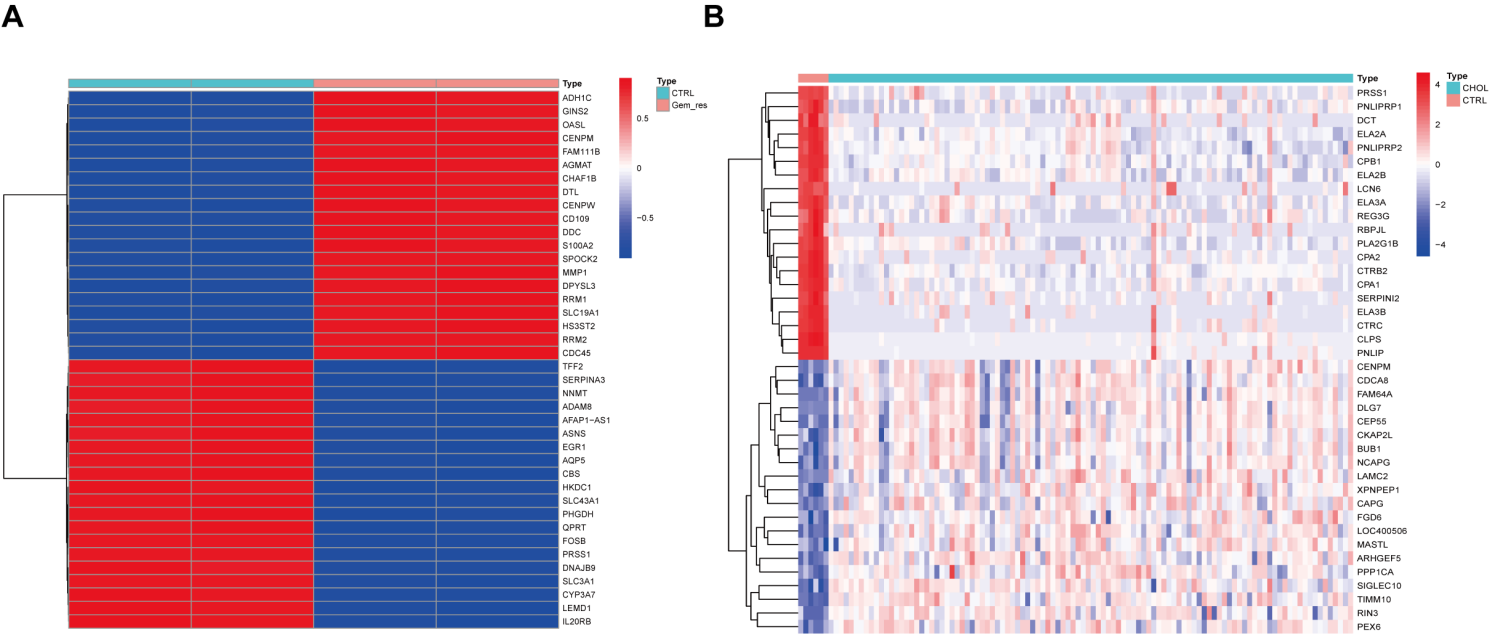


**Supplementary Figure 1. Differentially expressed gene analysis in gemcitabine-resistant cholangiocarcinoma. (A)** Heatmap showing DEGs identified in gemcitabine-resistant cholangiocarcinoma cell lines. **(B)** Heatmap displaying DEGs in cholangiocarcinoma tissues compared to normal bile duct tissues.


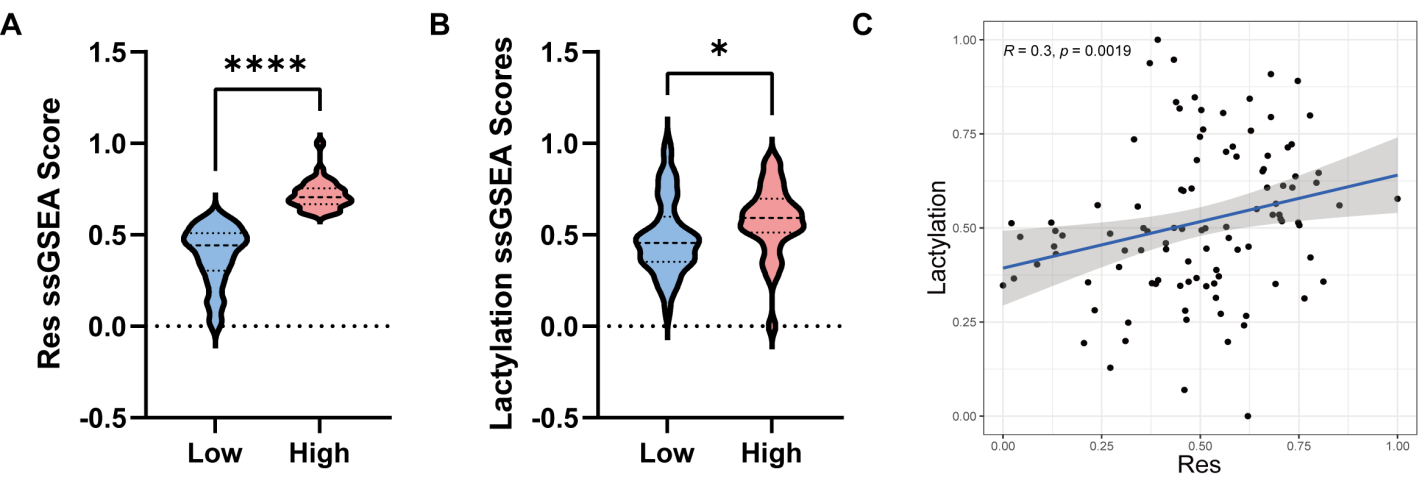


**Supplementary Figure 2.** **Validation of drug resistance-related molecular characteristics in an independent dataset. (A)** Violin plot confirming significantly higher drug resistance-related ssGSEA scores in the high group compared to the low group. **(B)** Violin plot showing significantly higher lactylation scores in the high group. **(C)** Spearman correlation analysis validating a significant positive correlation between drug resistance-related scores and lactylation scores.


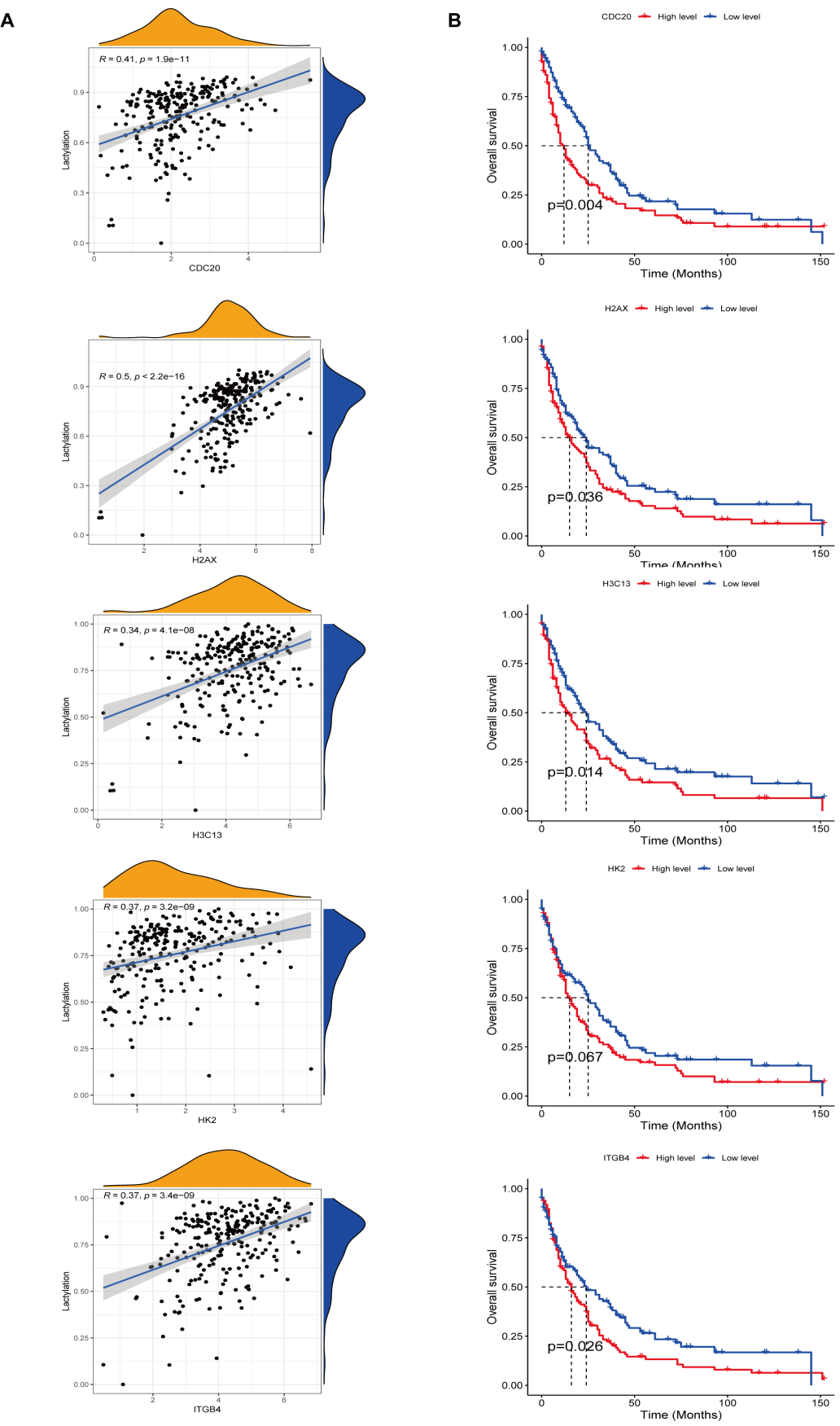


**Supplementary Figure 3.** **Correlation between feature gene expression and lactylation levels, as well as their impact on prognosis. (A)** Scatter plots illustrating the positive correlation between the expression levels of *CDC20*, *H2AX*, *H3C13*, *HK2*, and *ITGB4* and lactylation scores. **(B)** Kaplan-Meier survival analysis showing that high expression of these five genes is associated with poorer patient prognosis.


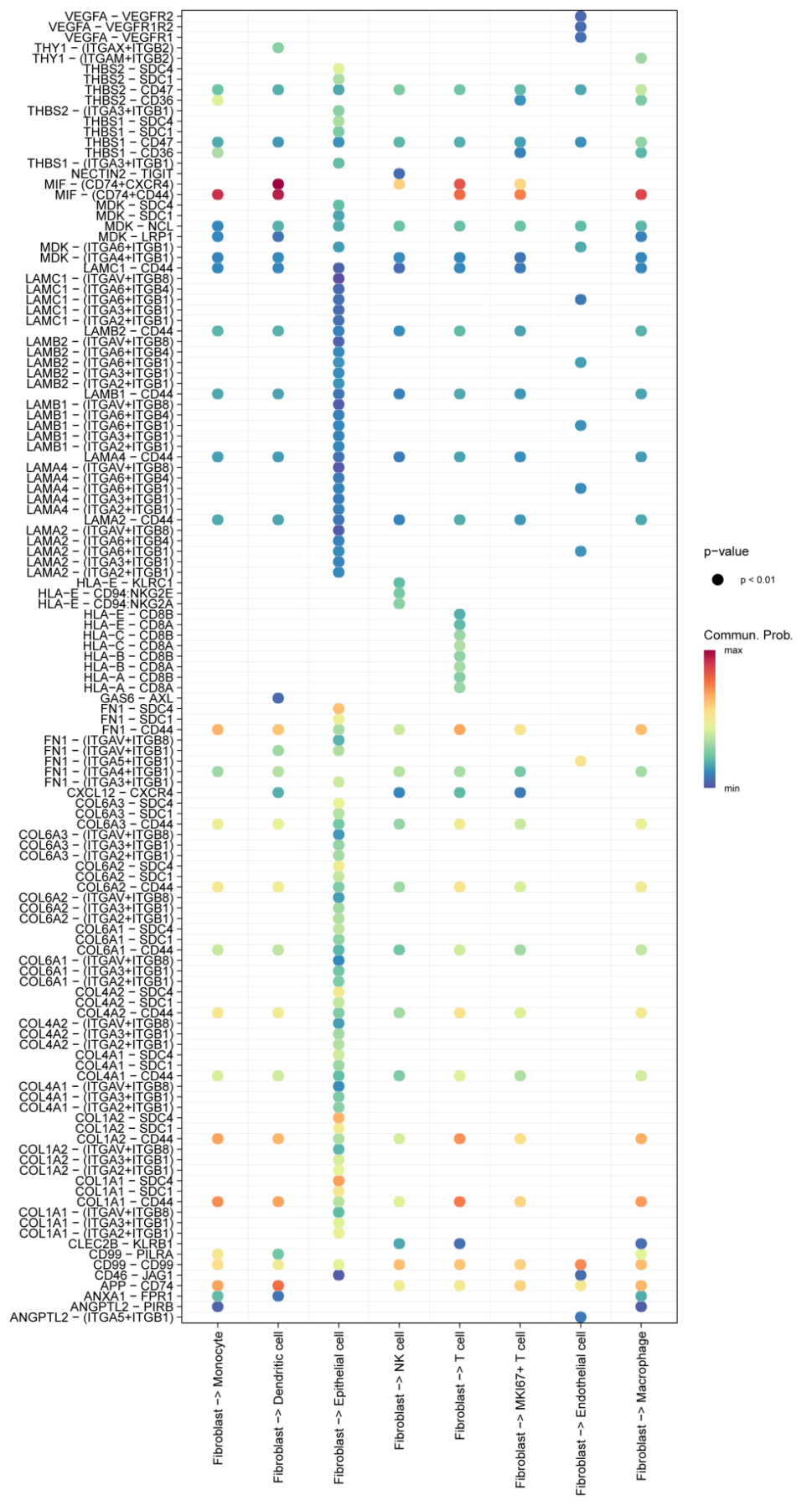


**Supplementary Figure 4.** **Receptor-ligand interactions between fibroblasts and other cell types in cholangiocarcinoma.**


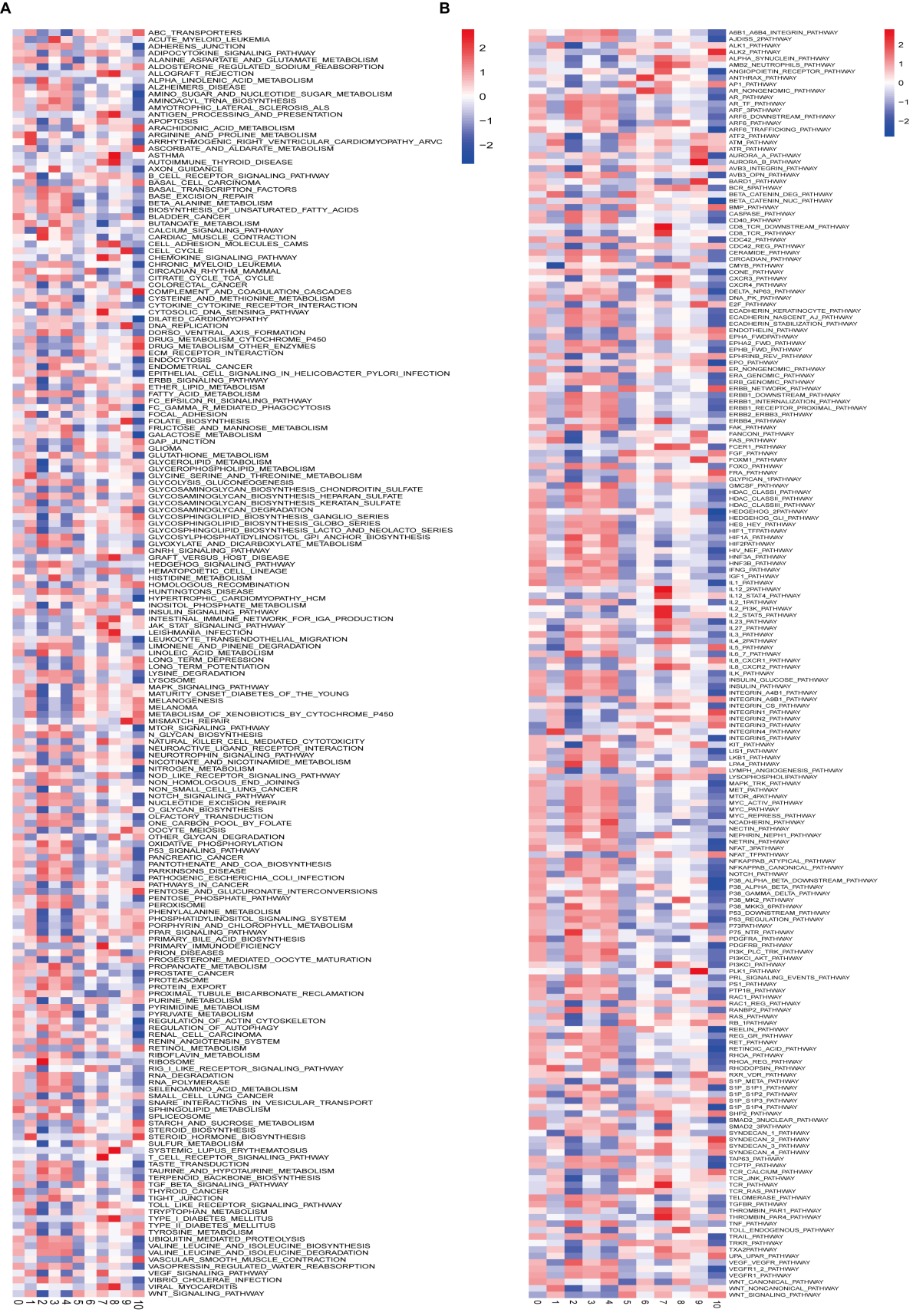


**Supplementary Figure 5.** **Pathway enrichment analysis of epithelial subpopulations. (A-B)** Heatmap displaying pathway activity across KEGG and PID gene sets for epithelial subpopulations.
